# In Vitro Evaluation of Probiotic Properties and Anti-Pathogenic Effects of *Lactobacillus* and *Bifidobacterium* Strains as Potential Probiotics

**DOI:** 10.3390/foods13142301

**Published:** 2024-07-22

**Authors:** Jaekoo Lee, Jaehyun Jo, Jungho Wan, Hanseul Seo, Seung-Won Han, Yoon-Jung Shin, Dong-Hyun Kim

**Affiliations:** 1 PB Business Department, NVP Healthcare Inc., Suwon 16209, Republic of Korea; jklee@nvp-healthcare.com (J.L.); jhjo@nvp-healthcare.com (J.J.); jhwan@nvp-healthcare.com (J.W.); hsseo@nvp-healthcare.com (H.S.); swhan@nvp-healthcare.com (S.-W.H.); 2 Department of Food Regulatory Science, Korea University, Sejong 30019, Republic of Korea; 3 Neurobiota Research Center, College of Pharmacy, Kyung Hee University, Seoul 02447, Republic of Korea; nayo971111@naver.com

**Keywords:** probiotics, gut health, probiotic properties, anti-pathogenic effects

## Abstract

Probiotics restore gut microbial balance, thereby providing health-promoting effects to the host. They have long been suggested for managing intestinal disorders caused by pathogens and for improving gut health. This study evaluated the probiotic properties and anti-pathogenic effects of specific probiotic strains against the intestinal pathogens *Staphylococcus aureus* and *Escherichia coli*. The tested strains—*Lactiplantibacillus plantarum* LC27, *Limosilactobacillus reuteri* NK33, *Lacticaseibacillus rhamnosus* NK210, *Bifidobacterium longum* NK46, and *Bifidobacterium bifidum* NK175—were able to survive harsh conditions simulating gastric and intestinal fluids. These strains exhibited good auto-aggregation abilities (41.8–92.3%) and ideal hydrophobicity (30.9–85.6% and 38.3–96.1% for xylene and chloroform, respectively), along with the ability to co-aggregate with *S*. *aureus* (40.6–68.2%) and *E*. *coli* (38.6–75.2%), indicating significant adhesion levels to Caco-2 cells. Furthermore, these strains’ cell-free supernatants (CFSs) demonstrated antimicrobial and antibiofilm activity against *S*. *aureus* and *E*. *coli*. Additionally, these strains inhibited gas production by *E*. *coli* through fermentative activity. These findings suggest that the strains tested in this study have potential as novel probiotics to enhance gut health.

## 1. Introduction

One of the most complex and abundant ecosystems is the human gut, comprising up to 10^13^–10^14^ microorganisms [1]. Many studies have demonstrated the significant roles played by gut microbiota in human health [2]. Probiotics, as living microorganisms, confer health benefits to the host by enhancing its intestinal microbial balance [3]. The application of probiotics has been frequently advised for the effective management of bowel disorders such as constipation and diarrhea, which arise from the disruption of microbial balance in the gut ecosystem [4]. Furthermore, probiotics have received considerable attention as health-promoting foods due to their various health benefits, such as the enhancement of immune function [5], maintenance of mental health and sleep quality [6], improvement of muscle strength [7], and alleviation of atopic dermatitis [8] in clinical studies.

Among various probiotic bacteria, *Lactobacillus* and *Bifidobacterium* are widely known genera due to their extensive history of use as probiotics [9]. These microorganisms, abundant in the human gut, are Gram-positive, non-spore-forming, rod-shaped, and catalase-negative. *Lactobacillus* and *Bifidobacterium* spp. are generally recognized as safe by the Food and Drug Administration and meet the qualified presumption of safety status established by the European Food Safety Authority [10]. The most studied probiotic strains include *L*. *plantarum*, *L*. *reuteri*, *L*. *rhamnosus*, *B*. *longum*, and *B*. *bifidum* [11]. Their potential as probiotics has been extensively reviewed, highlighting their effectiveness in managing various intestinal disorders such as lactose intolerance, infant gastroenteritis, diarrhea associated with rotavirus, antibiotic-induced diarrhea, and infant food allergies [3]. Furthermore, numerous research findings have demonstrated the ability of probiotics to regulate the gut microbiota and suppress pathogen growth in the gastrointestinal tract (GIT) [12,13]. Although the specific mechanisms mediating the inhibitory effects of probiotics on pathogens have yet to be fully elucidated, previous research suggests that probiotic bacteria can compete for attachment sites on the intestinal epithelial cells, secrete organic acids and antimicrobial compounds, and can stimulate and regulate the host’s immune system, thereby reducing intestinal microbial infections [14]. For *Lactobacillus* and *Bifidobacterium* strains to exert their proposed beneficial effects in the intestines, they must meet several prerequisites, including resistance to gastrointestinal conditions, adherence to mucus or gut epithelial cells, and antimicrobial activity through competition or the production of antimicrobial substances [15]. To this end, the World Health Organization has set up guidelines for evaluating probiotics, which include criteria for assessing efficacy and safety [16]. Furthermore, various in vitro assays have been developed and adopted as standards for selecting potential probiotics [17].

The overgrowth of *Staphylococcus aureus* and *Escherichia coli*, members of the intestinal flora, disrupts intestinal permeability by proliferating within the intestinal microbiota [18]. The alpha toxin, a significant virulence factor of *S. aureus*, disrupts intestinal integrity and leads to dysfunction in the intestinal epithelial barrier [19]. Enteropathogenic *E*. *coli* strains cause intestinal disorders such as Crohn’s disease, ulcerative colitis, and coeliac disease [20]. *S*. *aureus* and *E*. *coli* are well-known biofilm-forming pathogens, and the biofilms formed by these strains contribute to antibiotic resistance [21]. Treating infections caused by these biofilms can be challenging with antibiotics [22]. In this regard, there is considerable focus on finding strategies to eradicate biofilms or prevent their formation. The application of probiotics is one of the novel strategies for biofilm-forming *S*. *aureus* and *E*. *coli*. According to previous studies, certain probiotic strains have provided therapeutic advantages by suppressing the growth of *S*. *aureus* and *E*. *coli* and eradicating their biofilms [23,24].

Based on the above, the aim of this study was to evaluate the in vitro probiotic characteristics and anti-pathogenic effects of probiotic strains belonging to *Lactobacillus* and *Bifidobacterium* spp., isolated from kimchi or human feces for their potential application as dietary supplements. Therefore, their probiotic characteristics were evaluated by determining resistance to gastrointestinal conditions, cell surface properties, and adhesion to intestinal epithelial cells. Furthermore, to assess the anti-pathogenic effects, antimicrobial activity and antibiofilm activity against *S*. *aureus* and *E*. *coli* were investigated.

## 2. Materials and Methods

### 2.1. The Bacterial Strains and Culture Conditions

Probiotic strains (*Lactiplantibacillus plantarum* LC27, Limosilactobacillus reuteri NK33, *Lacticaseibacillus rhamnosus* NK210, *Bifidobacterium longum* NK46, and *Bifidobacterium bifidum* NK175), and ATCC indicator strains (*S. aureus* ATCC 25923 and *E. coli* ATCC 25922) used in the present study were obtained from the Korea Culture Center of Microorganisms (KCCM, Seoul, Republic of Korea) and the Korean Collection for Type Cultures (KCTC, Daejeon, Republic of Korea). Probiotic strains were cultured in De Man, Rogosa, and Sharpe broth (MRS; Difco Laboratories, Detroit, MI, USA) at 37 °C under anaerobic conditions. Meanwhile, *S. aureus* ATCC 25923 and *E. coli* ATCC 25922 were cultured in brain heart infusion broth (BHI; Difco Laboratories) and Luria-Bertani broth (LB; Difco Laboratories), respectively, at 37 °C under aerobic conditions.

### 2.2. Cell-Free Supernatants Preparation

Cell-free supernatants (CFSs) were prepared following a previous study [25]. The probiotic cultures were centrifuged at 4000× *g* for 20 min. The supernatant was collected and filtered through 0.22 µm Millipore filters to sterilize. The resulting CFSs were then used in vitro and stored at −20 °C for further study.

### 2.3. Tolerance to Simulated Gastrointestinal Conditions

The resistance of the probiotic strains to gastrointestinal conditions was assessed by examining their viability after exposure to simulated gastric fluid (SGF) and simulated intestinal fluid (SIF), as described by Lee et al. [25], with minor modifications. The viability of tested probiotic strains was assessed following 2 h of exposure to SGF, consisting of phosphate-buffered saline (PBS; pH 2.5) with 0.3% pepsin (Sigma, St. Louis, MO, USA), at 37 °C. Subsequently, after 2.5 h, viability was measured in SIF, comprising PBS (pH 7.4) with 1% (*w*/*v*), pancreatin (Wako, Osaka, Japan), and 1% (*w*/*v*) bile salt (Difco Laboratories) at 37 °C. The plate counting method was used to determine bacterial viability, and the survival rate was calculated using the following Equation (1):

(1) $$Survival\;rate\;\left(\%\right)=\left(Log\;N/Log\;N_{0}\right)\times 100$$

where *Log N*_0_ and *Log N* are the logarithm of the number of viable cells before and after exposure to the test condition, respectively.

### 2.4. Auto-Aggregation and Co-Aggregation Assay

Auto-aggregation and co-aggregation assays were conducted according to the method described by Balakrishna [26], with slight modifications. Briefly, the probiotic strains were cultured in MRS broth at 37 °C for 18 h under anaerobic conditions. Bacterial cells were harvested by centrifugation at 4000× *g* for 20 min, washed twice with PBS, and then resuspended in the same buffer to adjust to approximately 1 × 10^8^ colony-forming units (CFU)/mL viable counts. The cell suspension was vortexed for 30 s to mix and incubated at 37 °C for 5 h. The absorbance of the upper suspension was measured at 600 nm. The auto-aggregation percentage was calculated using the following Equation (2):

(2) $$Auto-aggregation\;\left(\%\right)=\left(1-A_{t}/A_{0}\right)\times 100$$

where *A_t_* represents the absorbance at time *t* = 5 h and *A*_0_ is the absorbance at *t* = 0.

The bacterial suspension was prepared in a similar manner for the co-aggregation assay. Each cell suspension of the probiotic strains and indicator strains was mixed together in equal volumes by vortexing for 30 s. Multiple control tubes were prepared, each containing 4 mL of an individual bacterial cell suspension. After incubating at 37 °C for 5 h, the absorbance of the suspension was measured at 600 nm. The co-aggregation percentage was calculated using the following Equation (3):

(3) $$Co-aggregation\;\left(\%\right)=\left[1-2A_{mix}/\left(A_{probio}+A_{indicat}\right)\right]\times 100$$

where *A_probio_* and *A_indicat_* represent the absorbance of individual bacterial suspensions in the control tubes and *A_mix_* represents the absorbance of the mixtures after 5 h.

### 2.5. Bacterial Adhesion to Solvents Assay

To assess bacterial cell surface properties, bacterial adhesion to solvent (BATS) assay was conducted according to the method described by Kumari et al. [27], with minor modifications. In this study, the adherence of probiotic strains to three different solvents was tested: xylene (Duksan Pure Chemical Co., Ltd., Ansan, Republic of Korea), a non-polar solvent; chloroform (Samchun Pure Chemical Co., Ltd., Seoul, Republic of Korea), a monopolar and Lewis-acid solvent; and ethyl acetate (Samchun Pure Chemical Co., Ltd., Detroit, MI, USA.), a monopolar and Lewis-base solvent. Briefly, bacterial cells were suspended at approximately 1 × 10^8^ CFU/mL in PBS (pH 7.4). One milliliter of each solvent was added to 3 mL of the bacterial cell suspension. After vortexing for 2 min, the mixture was incubated at 37 °C for 5 min to allow for phase separation. The optical density of the aqueous phase was measured at 600 nm using a microplate reader (Bio-Tek, Winooski, VT, USA). The affinities to solvents with different physicochemical characteristics were calculated using the following Equation (4):

(4) $$BATS\;\left(\%\right)=\left(1-A_{t}/A_{0}\right)\times 100$$

where *A*_0_ and *A_t_* represent the absorbance before and after extractions with different solvents, respectively. Strains were categorized into three groups following Zhang et al. [28]: low (0–35%), intermediate (36–70%), and high (71–100%).

### 2.6. Cytotoxicity Assay and Adhesion Determination

Caco-2 (Korea Cell Line Bank, Seoul, Republic of Korea) was cultured in Dulbecco’s Modified Eagle’s Medium (Gibco, Grand Island, NY, USA) supplemented with 10% heat-inactivated fetal bovine serum (Gibco) and 1% antibiotic–antimycotic solution (Gibco) at 37 °C in 5% CO_2_ humidified atmosphere. The cytotoxicity of the probiotic strains was assessed using the Quanti-LDH PLUS Cytotoxicity Assay Kit (Biomax, Seoul, Republic of Korea) according to the manufacturer’s instructions. Briefly, Caco-2 cells were plated in 96-well plates at a density of 1 × 10^4^ cells per well. The probiotic strains were then treated with 10^9^ CFU/mL for 24 h. Cell lysis buffer was employed as a positive control. Subsequently, 50 μL of supernatants, from which cells were removed, were mixed with 50 μL of substrate mix and incubated for 30 min in the dark. The absorbance was measured at 490 nm, and *Cytotoxicity* was determined using the following Equation (5):

(5) $$Cytotoxicity\;\left(\%\right)=\left[\left(Sample-Low\;control\right)/\left(High\;control-Low\;control\right)\right]\times 100$$

where *Sample* represents the absorbance of the supernatant from Caco-2 cells treated with the probiotic strains, *Low control* represents the absorbance of the supernatant from cells without treatment, and *High control* represents the absorbance from cells after lysis.

The adhesion capability of the probiotic strains to the Caco-2 cells, an intestinal epithelial cell line, was assessed using the method described by Lee et al. [29]. Caco-2 cells were plated at a density of 1 × 10^5^ cells/well in 12-well plates and incubated at 37 °C for 24 h. One milliliter of bacterial cell suspension (1 × 10^8^ CFU/mL) was added to each well and incubated at 37 °C for 2 h in a humidified atmosphere with 5% CO_2_. The cells were then rinsed three times with PBS to remove non-attached bacterial cells, followed by treatment with a 1% (*v*/*v*) Triton X-100 solution (Daejung Chemical Co., Ltd., Shiheung, Republic of Korea) to release attached bacterial cells. The number of bacteria adhered to the cells was assessed using the plate count method. The *Adhesion ability* was calculated using the following Equation (6):

(6) $$Adhesion\;ability\;\left(\%\right)=\left[adherent\;cells\;\left(\log CFU/mL\right)/initial\;cells\;\left(\log CFU/mL\right)\right]\times 100$$

### 2.7. Antimicrobial Activity

The antimicrobial activity of the probiotic strains against the indicator strains was assessed using the agar overlay method, as previously described by Halder et al. [30], with some modifications. Briefly, 10 μL of bacterial cell suspension containing approximately 1 × 10^8^ CFU/mL was spotted onto MRS agar and then incubated at 37 °C for 24 h under anaerobic conditions. Before overlaying with the indicator strains, the agar plates were exposed to chloroform vapor to kill the viable probiotic strains. Thereafter, the MRS agar plates harboring spotted probiotic strains were overlaid with soft BHI agar or soft LB agar (0.7% agar) containing approximately 1 × 10^8^ CFU of the indicator strains. After the overlaid agar medium solidified, the plates were incubated at 37 °C for 24 h, and the inhibition zone was measured. The width of the clear zone (*R*) was determined and interpreted according to the method outlined by Carasi et al. [31]: *R* = (*d*_Inhib_ − *d*_Spot_)/2, where *d*_Inhib_ represents the diameter of the zone around the spot and *d*_Spot_ is the diameter of the probiotic strain spot. Inhibition scores were expressed as no (*R* < 2 mm), low (2 mm < *R* < 5 mm), and high (*R* > 6 mm) inhibition, respectively.

### 2.8. Minimum Inhibitory Concentrations of the Cell-Free Supernatants 

The minimum inhibitory concentrations (MICs) of the CFSs of the tested probiotic strains against *S*. *aureus* and *E*. *coli* were evaluated using the microtiter plate assay [24]. In brief, various concentrations of the CFSs of the probiotic strains were prepared in the following range: 10, 20, 30, 40, and 50 mg/mL. Indicator strains at a concentration of 10^6^ CFU/well were inoculated in each growth medium containing the various concentrations of the CFSs. After incubation at 37 °C for 24 h under aerobic conditions, bacterial growth was assessed by measuring the absorbance at 600 nm. The MIC was defined as the lowest quantity of the CFSs at which no visible growth was observed.

### 2.9. Biofilms Eradication and Inhibition Assay

Biofilm formation was determined using crystal violet staining, as described by Zamani et al. [32]. To confirm the effect of CFSs on the eradication of biofilms established by both *S*. *aureus* and *E*. *coli*, pathogenic bacteria were cultured at 37 °C for 24 h in 96-well plates. Then, the medium was removed from each well, and the established biofilms were gently washed twice using PBS to avoid disrupting the biofilm. The CFSs from the tested probiotic strains were added to each well and incubated at 37 °C for a further 24 h. In the control wells, MRS broth without probiotic strains was used instead of CFSs. Subsequently, the plates were rinsed twice with PBS, and 0.1% crystal violet solution was added to the wells. After incubation for 15 min, the plates were washed and subsequently allowed to dry. Finally, the fixed crystal violet within the biofilm was dissolved in a 30% acetic acid solution. The absorbance at 550 nm was recorded, and the biofilm formation rate was calculated using the following Equation (7):

(7) $$Biofilms\;eradication\;or\;inhibition\;\left(\%\right)=\left(1-{OD}_{sample}/{OD}_{control}\right)\times 100$$

A similar method was employed to confirm the effect of CFSs on the inhibition of biofilm formation. Briefly, pathogenic bacteria were cultured with the CFSs in 96-well plates. After incubation at 37 °C for 24 h, the same crystal violet method and calculation steps were employed as described above to determine the percentage of biofilm inhibition.

### 2.10. Inhibition of Gas Production

The probiotic strains tested in this study were assessed for their capacity to inhibit gas production resulting from the fermentative action of *E*. *coli*, as described by Jang et al. [23]. Briefly, 50 μL of *E*. *coli* culture, at approximately 1 × 10^8^ CFU/mL, was inoculated into the top third layer of the LB agar, composed of 5 mL per tube. Subsequently, 50 μL of each probiotic culture was added to 3 mL of soft MRS agar (0.7% agar). The mixture was vortexed for homogenization and immediately poured onto the LB agar layer in tubes with *E*. *coli*, followed by aerobic incubation at 37 °C for 24 h. Negative controls consisted of LB agar inoculated with *E*. *coli* and MRS agar without the probiotic strains.

### 2.11. Statistical Analysis

Data analyses were conducted using SPSS software version 26 (IBM Inc., Armonk, NY, USA). Differences between groups were assessed for statistical significance through one-way analysis of variance (ANOVA), followed by Tukey’s post hoc test with a significance level set at *p* < 0.05. All results are expressed as the mean ± standard deviation (SD) from three independent experiments.

## 3. Results and Discussion

### 3.1. Resistance of the Probiotic Strains to Simulated Gastrointestinal Conditions

When transiting through the GIT, probiotic microorganisms encounter several challenges, such as the low pH conditions of the stomach, bile salts, and digestive enzymes of the intestinal tract [33]. To provide health benefits to the host, they need to withstand these harsh conditions [34]. In this study, the GIT resistance of probiotic strains was examined under an in vitro simulated continuous digestion model containing SGF and SIF (Table 1). Among the tested strains, *L*. *reuteri* NK33 showed the highest survival rate (99.3 ± 0.1%), followed by *L*. *rhamnosus* NK210 (89.5 ± 0.4%), *L*. *plantarum* LC27 (42.0 ± 2.0%), *B*. *longum* NK46 (40.8 ± 1.4%), and *B*. *bifidum* NK175 (30.7 ± 0.1%). After exposure to SGF for 2 h, the cell viability decreased in all tested strains except for *L*. *reuteri* NK33. Conversely, there was a minimal decrease in cell viability following exposure to SIF supplemented with 1% (*w*/*v*) bile salts and 1% (*w*/*v*) pancreatin. Consistent with these findings, previous studies have reported that the survival rates of certain strains of *Lactobacillus* and *Bifidobacterium* notably declined following exposure to SGF while exhibiting minimal decrease after exposure to SIF [25,35,36]. On the other hand, some studies have documented that certain probiotic strains exhibit outstanding survival rates under gastric acidic conditions [24,37]. However, in these studies, the bacteria were cultured in a medium or peptone water to minimize damage. In this study, we assessed the survival of probiotic strains in simulated gastrointestinal fluid without any protective compounds and verified that their survival aligned with that of previous studies [25,38]. These findings suggest that the tested probiotic strains could withstand and persist in gastrointestinal conditions.

### 3.2. Auto-Aggregation and Co-Aggregation Activity

**Figure 1:**
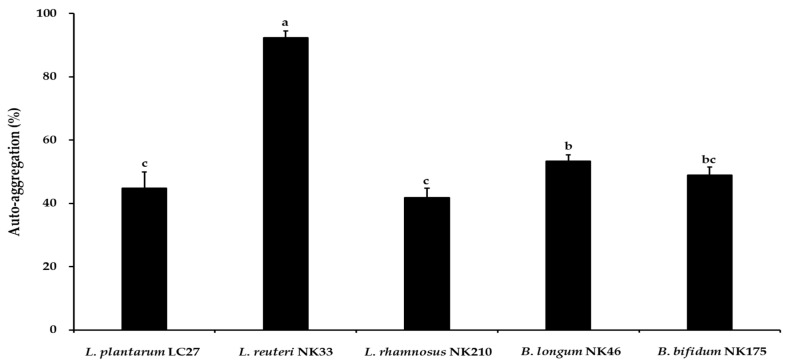
Auto-aggregation of the tested probiotic strains. Data are shown as the mean ± standard deviation (SD) of three independent experiments (*n* = 3). Different letters on the column denote significant differences between means at *p* < 0.05, as determined by Tukey’s post hoc test.

The auto-aggregation of probiotic strains increases their adhesion to intestinal epithelial cells and inhibits the colonization of pathogenic bacteria [39]. In this study, auto-aggregation of the putative probiotic strains was evaluated using an aggregation visual assay, and the results are shown in Figure 1. The auto-aggregation values of probiotic strains ranged between 41.8% and 92.3% at 37 °C. *L*. *reuteri* NK33 showed the highest auto-aggregation ability (92.3 ± 2.2%), followed by *B*. *longum* NK46 (53.3 ± 1.9%), *B*. *bifidum* NK175 (48.9 ± 2.5%), *L*. *plantarum* LC27 (44.7 ± 5.2%), and *L*. *rhamnosus* NK210 (41.8 ± 2.9%). Previous studies indicated that the auto-aggregation values of commercial probiotic strains, *L. rhamnosus* GG, *L*. *rhamnosus* GR-1, and *L*. *acidophilus* La-5, were 41.4 ± 3.3%, 15.2 ± 0.6%, and 15.9 ± 1.1%, respectively [40,41]. Furthermore, certain strains of *Lactobacillus* and *Bifidobacterium* spp. displayed low auto-aggregation abilities ranging from 11.5% to 29.0% [39].

**Figure 2:**
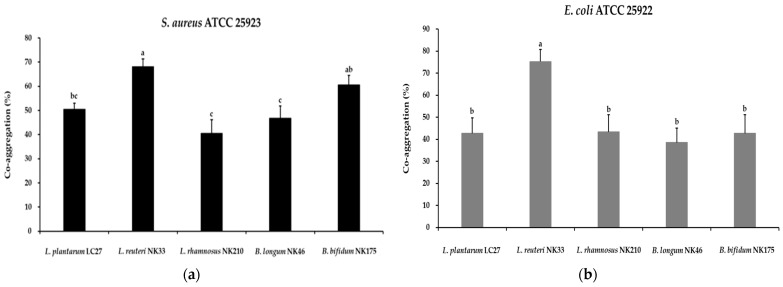
Co-aggregation of the tested probiotic strains and pathogens *S*. *aureus* ATCC 25923 (**a**) and *E*. *coli* ATCC 25922 (**b**) after 24 h of incubation at 37 °C. Data are shown as the mean ± SD of three independent experiments (*n* = 3). Different letters on the column denote significant differences between means at *p* < 0.05, as determined by Tukey’s post hoc test.

The co-aggregation abilities of probiotic strains could disrupt the ability of pathogenic strains to infect the host and hinder the colonization of foodborne pathogens [40]. This results in the therapeutic benefits of probiotics for infections. The co-aggregation abilities of probiotic strains with tested pathogens (*S*. *aureus* ATCC 25923 and *E*. *coli* ATCC 25922) are presented in Figure 2. In the present study, all strains exhibited co-aggregation with the tested pathogens, and notably, *L*. *reuteri* NK33 showed better co-aggregation than other probiotic strains. Previous studies reported that the differences in co-aggregation ability of probiotic strains depended on the specific probiotic and pathogenic strains [27,40], which is similar to the results of the present study.

Significant correlations (*p* < 0.01) were found between the auto-aggregation and co-aggregation abilities of the five probiotic strains, as shown in Table 2. This is consistent with previous studies, which reported that strains exhibiting greater auto-aggregation displayed high co-aggregation against pathogens [42,43,44]. Therefore, auto-aggregation properties, along with the co-aggregation ability against potential pathogens, can help identify which strains possess the most promising potential to thrive and exert beneficial effects within the gut microbiota.

### 3.3. Cell Surface Hydrophobicity

**Figure 3:**
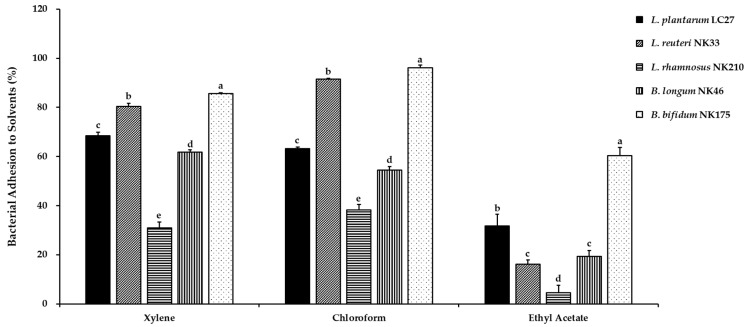
Cell surface hydrophobicity of the tested probiotic strains in three different solvents (xylene, chloroform, and ethyl acetate). Data are shown as the mean ± SD of three independent experiments (*n* = 3). Different letters on the column denote significant differences between means at *p* < 0.05, as determined by Tukey’s post hoc test.

In this study, the physicochemical properties of the bacterial cell surface of the tested probiotic strains were characterized using bacterial adhesion to different solvents, including xylene, chloroform, and ethyl acetate, and the results are presented in Figure 3. To assess cell surface hydrophobicity or hydrophilicity, the affinity for xylene was evaluated. The results indicated that *L*. *reuteri* NK33 (80.4 ± 1.3%) and *B*. *bifidum* NK175 (85.6 ± 0.4%) exhibited high hydrophobicity, while *L*. *rhamnosus* NK210 (30.9 ± 2.3%) showed low hydrophobicity. The bacterial cell surface exhibits both hydrophobic and hydrophilic properties due to variances in the makeup of surface polymers involved in hydrophobic interactions. Hydrophobic properties result from proteins, teichoic acid, and lipoteichoic acid on the bacterial cell wall, while polysaccharides enhance hydrophilic properties [40]. Furthermore, we performed the bacterial adhesion to chloroform and ethyl acetate assay to assess the electron donor and acceptor properties of the bacterial cell surface. The highest affinities with chloroform, similar to the affinity for xylene, were observed in *L*. *reuteri* NK33 (91.6 ± 0.3%) and *B*. *bifidum* NK175 (96.1 ± 1.1%), suggesting their strong electron donor properties. When compared to xylene and chloroform, the affinities with ethyl acetate were significantly decreased in all tested probiotic strains, suggesting these strains possess non-acidic and poor electron acceptor characteristics.

A previous study reported a relationship between auto-aggregation property and BATS [44]. In this study, no statistically significant correlations were observed between the two properties, except for chloroform affinity, for the tested probiotic strains belonging to different genera (Table 2). The results of the present study are consistent with the work of Vlková et al. [45], who reported no correlation between the auto-aggregation property of *Bifidobacterium* spp. and their hydrophobicity. Among the three *Lactobacillus* species studied here, auto-aggregation showed a high correlation with affinity to xylene (*r* = 0.715, *p* < 0.05) and chloroform (*r* = 0.899, *p* < 0.01), as shown in Figure S1. Cell surface hydrophobicity is one of several mechanisms contributing to the adherence of probiotic strains to gut epithelial cells [27]. However, no statistically significant correlations were observed between bacterial surface hydrophobicity and the ability of adhesion to Caco-2 cells in this study (Table 2). Consistent with these findings, previous studies indicated that strains with strong hydrophobicity did not consistently adhere effectively to intestinal epithelial cells. Bacteria adjust their surface hydrophobicity in response to environmental variations such as pH, ionic strength, or surface structure [46,47].

### 3.4. Adhesion Ability of the Probiotic Strains

**Figure 4:**
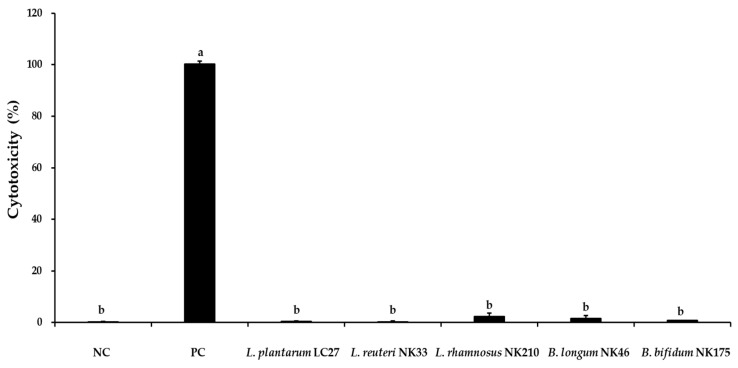
Evaluation of cytotoxicity of the tested probiotic strains against Caco-2 cells. Data are shown as the mean ± SD of three independent experiments (*n* = 3). Different letters on the column denote significant differences between means at *p* < 0.05, as determined by Tukey’s post hoc test. NC: negative control; PC: positive control.

**Figure 5:**
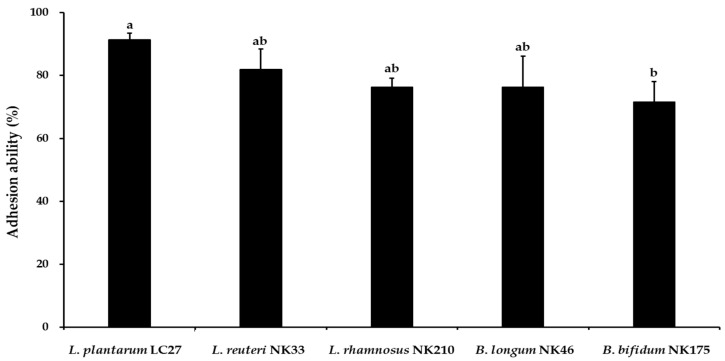
Adhesion property of the tested probiotic strains to the intestinal Caco-2 cell line. The adhesion ability is determined by the ratio of the initially added bacteria to the number of adhered bacterial cells. Data are shown as the mean ± SD of three independent experiments (*n* = 3). Different letters on the column denote significant differences between means at *p* < 0.05, as determined by Tukey’s post hoc test.

Adherence to intestinal epithelial cells is considered a crucial criterion for selecting potential probiotic strains [43]. Moreover, adhesion capability helps probiotics extend their survival in the GIT and enhance their interactions with the host [48]. Prior to the adhesion capability test, the cytotoxicity of the tested probiotic strains against Caco-2 cells was evaluated to ensure their safety. Following a 24 h incubation at a concentration of 1 × 10^9^ CFU/mL of the tested probiotic strains, no cytotoxic effects were detected on Caco-2 cells (Figure 4). For the adhesion capability test, probiotic strains were inoculated at a concentration of 1 × 10^8^ CFU/mL, and the results are shown in Figure 5. The most adhesive strains were *L*. *plantarum* LC27 (91.2 ± 2.2%) and *L*. *reuteri* NK33 (81.9 ± 6.5%), followed by *L*. *rhamnosus* NK210 (76.3 ± 2.8%), *B*. *longum* NK46 (76.3 ± 9.9%), and *B*. *bifidum* NK175 (71.5 ± 6.5%). There were no significant differences observed among the strains regarding their adhesion percentage to Caco-2 cells, except for *B*. *bifidum* NK175. Patrone et al. [49] reported that *L*. *rhamnosus* GG had an adhesion ability of approximately 78% to Caco-2 cells. Furthermore, commercial probiotic strains such as *Lactobacillus* spp. and *Bifidobacterium* spp. demonstrated robust adhesion abilities (>70%) to intestinal epithelial cells [50,51]. Therefore, these findings suggest that all the strains tested in this study have the potential to adhere to and colonize intestinal epithelial cells.

### 3.5. Antimicrobial Activity of the Probiotic Strains

The antimicrobial activity of probiotics is considered a crucial trait for restricting the proliferation of pathogenic bacteria, thereby maintaining a healthy microbial balance in the gut. Probiotics, primarily lactobacilli and bifidobacteria, can inhibit pathogen growth by producing bioactive molecules such as organic acids, hydrogen peroxide, and bacteriocins [30]. To assess whether the extracellular products of probiotic strains tested in this study inhibit the growth of enteric pathogens, antimicrobial activity tests were conducted using Gram-positive (*S*. *aureus* ATCC 25923) and Gram-negative (*E*. *coli* ATCC 25922) pathogenic bacteria. As observed in Figure S2 and Table 3, the results of this study revealed that all probiotic strains exhibited remarkable antagonistic activity against the tested pathogenic bacteria, with the inhibition profile varying depending on the strains, consistent with previous studies [30]. Furthermore, according to the *R* value, all probiotic strains exhibited superior antagonistic activity against *E*. *coli* ATCC 25922 compared to *S*. *aureus* ATCC 25923.

Additionally, the CFSs from probiotic strains were evaluated for their antimicrobial activity against pathogens using MIC values, as presented in Table 4. All tested strains, except for *L*. *rhamnosus* NK210, exhibited the same MIC values for *S*. *aureus* and *E*. *coli*. The CFS of *L*. *plantarum* LC27 exhibited the most effective antimicrobial activity against *S*. *aureus* and *E*. *coli*, with MIC values of 20 mg/mL, respectively. Consistent with the findings of the present study, previous research has demonstrated that CFSs from *L*. *plantarum*, *L*. *reuteri*, *L*. *rhamnosus*, and *B*. *longum* exhibit antimicrobial activity against *S*. *aureus* and *E*. *coli* [52,53,54]. These findings suggest that the metabolites produced by probiotic strains significantly contribute to their antimicrobial potential, and the use of probiotic bacteria with the ability to suppress pathogenic gut microflora could potentially benefit the host’s health.

### 3.6. Antibiofilm Activity of the Probiotic Strains

**Figure 6:**
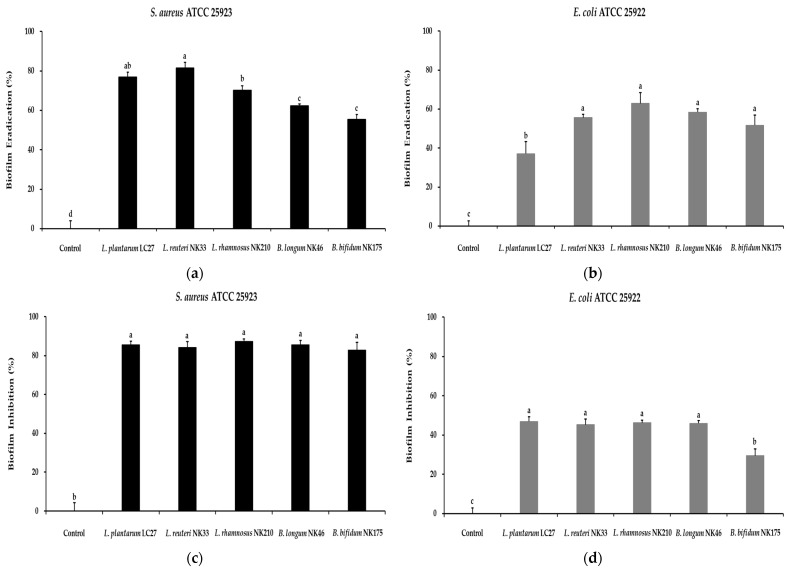
Antibiofilm activity of the tested probiotic strains against pathogens. Eradication effects of the tested probiotic strains on biofilms formed by *S*. *aureus* ATCC 25923 (**a**) and *E*. *coli* ATCC 25922 (**b**). Inhibitory effects of the tested probiotic strains on biofilm formation by *S*. *aureus* ATCC 25923 (**c**) and *E*. *coli* ATCC 25922 (**d**). Data are shown as the mean ± SD of three independent experiments (*n* = 3). Different letters on the column denote significant differences between means at *p* < 0.05, as determined by Tukey’s post hoc test.

Biofilms, complex bacterial aggregates, consist of a microbial community and a self-produced extracellular matrix [23]. Pathogens can withstand harsh environments through biofilm formation. Dispersion of biofilms promotes pathogen spread, contributing to food contamination, spoilage, and chronic infections [55]. Consequently, new strategies are needed to remove and prevent biofilm formation by pathogens. The efficacy of the CFSs from probiotic strains in eradicating biofilms formed by indicator pathogens was evaluated using a crystal violet assay. Biofilms were significantly reduced after treatment with CFSs compared to the untreated control group (Figure 6a). Biofilms formed by *S*. *aureus* were eliminated by 55.3–81.5%, while eradication of *E*. *coli* biofilms ranged from 37.2% to 62.9% when exposed to CFSs. Consistent with these findings, a previous study reported that probiotic mixtures containing *Lactobacillus* and *Bifidobacterium* spp. exhibit higher eradication of *S*. *aureus* biofilms compared to those formed by *E*. *coli* [23]. Additionally, Zamani et al. [32] reported that CFSs containing digestive enzymes aid in the eradication of preformed biofilms by digesting extracellular compounds produced by biofilm-forming cells. Furthermore, pathogenic bacteria were co-incubated with CFSs to confirm their effect on inhibiting biofilm formation. Significant inhibition in biofilm formation was observed in CFSs-treated wells, with no significant differences observed between the strains, except for *B*. *bifidum* NK175, in terms of inhibiting biofilm formation by *S*. *aureus* and *E*. *coli* (Figure 6b). CFSs suppressed *S*. *aureus* biofilm formation by over 80% and inhibited *E*. *coli* biofilm formation within the range of 29.5% to 46.9%. Consistent with our findings, previous studies have demonstrated that CFSs from certain lactic acid bacteria inhibit the formation of *S*. *aureus* or *E*. *coli* biofilms [22,32,56]. These results suggest that the probiotic strains used in the present study may represent a promising alternative approach for controlling pathogen biofilm formation in the food industry.

### 3.7. Inhibition of Gas Production by E. coli from the Probiotic Strains

**Figure 7:**
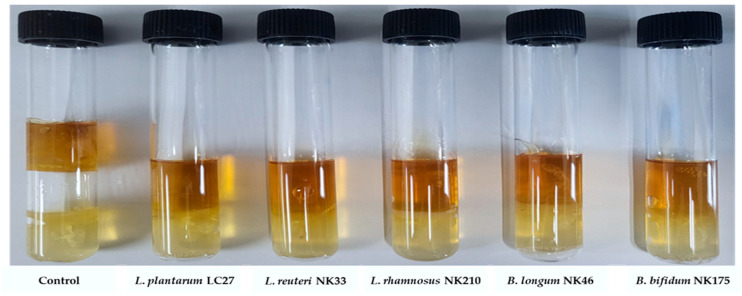
Inhibitory effects of the tested probiotic strains on gas production by *E*. *coli* ATCC 25923. The tube inoculated only with *E*. *coli* was used as control group.

Some patients with gastrointestinal disorders experience abdominal bloating, potentially due to intestinal gas production by intestinal bacteria, including pathogens [23]. To evaluate whether probiotic strains alleviate pathogen-induced abdominal bloating, the inhibition of gas production was assessed. *E*. *coli* was chosen due to its inherent capacity to generate significant amounts of hydrogen gas through fermentative activity [57]. All tested probiotic strains inhibited gas production by *E*. *coli*, whereas gas production was observed in the control tube inoculated only with *E*. *coli* (Figure 7). Consistent with these findings, Jang et al. [23] reported that probiotic mixtures containing *Lactobacillus* and *Bifidobacterium* spp. inhibited gas production by *E*. *coli*. Furthermore, certain probiotic strains, including *L*. *plantarum*, *L*. *rhamnosus*, and *B*. *longum*, inhibited gas production by *Clostridium butyricum* [58]. Although further studies are needed to determine if gas production by other bacteria can be inhibited, our findings suggest that the tested probiotic strains can alleviate abdominal bloating caused by gas-producing pathogens.

## 4. Conclusions

Probiotics have gained significant attention in the functional food market due to growing evidence from clinical trials demonstrating a variety of beneficial effects. For probiotics to provide their beneficial effects, they need to meet various desired properties, but finding probiotic strains that possess all the ideal characteristics is challenging. Probiotic strains tested in this study (*Lactiplantibacillus plantarum* LC27, *Limosilactobacillus reuteri* NK33, *Lacticaseibacillus rhamnosus* NK210, *Bifidobacterium longum* NK46, and *Bifidobacterium bifidum* NK175) exhibited several essential probiotic properties. Among these strains, *L*. *reuteri* NK33 proved to be best—it shows high resistance to gastrointestinal conditions, robust auto-aggregation and co-aggregation abilities, good cell surface hydrophobicity, and effective adhesion to intestinal epithelial cells. Furthermore, CFSs from these probiotic strains effectively inhibited the growth of *S*. *aureus* and *E*. *coli*, eradicated their biofilms, and prevented the formation of new biofilms, and these strains also inhibited gas production by *E*. *coli*. Therefore, these findings suggest that incorporating these probiotic strains into dietary supplements could effectively inhibit *S*. *aureus* and *E*. *coli*, both of which can trigger intestinal disorders. However, further studies, such as in vivo and clinical trials, are required to confirm their inhibitory effects against intestinal pathogens when ingested.

## Figures and Tables

**Table 1:** Survival rate of the tested probiotic strains under simulated gastric and intestinal conditions.

Strains	Initial Counts (log CFU/mL)	SGF (log CFU/mL)	SIF (log CFU/mL)	Survival Rate (%)
*L*. *plantarum* LC27	7.04 ± 0.08	2.98 ± 0.09 *	2.96 ± 0.11 *	42.04 ± 2.01 ^c^
*L. reuteri* NK33	7.52 ± 0.02	7.48 ± 0.13	7.47 ± 0.03 *	99.27 ± 0.12 ^a^
*L. rhamnosus* NK210	7.33 ± 0.02	6.72 ± 0.04 *	6.56 ± 0.02 *	89.54 ± 0.38 ^b^
*B. longum* NK46	7.84 ± 0.04	3.24 ± 0.11 *	3.20 ± 0.13 *	40.82 ± 1.41 ^c^
*B. bifidum* NK175	7.15 ± 0.03	2.73 ± 0.05 *	2.19 ± 0.01 *	30.65 ± 0.06 ^d^

Data are presented as the mean ± SD of three independent experiments (*n* = 3). Asterisks (*) indicate significant differences from the initial counts (*p* < 0.05), determined by Student’s *t*-test. Different letters in the same column indicate significant differences between means at *p* < 0.05 based on Tukey’s post hoc test. SGF: simulated gastric fluid; SIF: stimulated intestinal fluid.

**Table 2:** Results of Pearson correlation analysis for the cell surface properties and adhesion properties.

	Adhesion	Auto-Aggregation	Co-Aggregation		Hydrophobicity		
			*S*. *aureus* ATCC 25923	*E*. *coli* ATCC 25922	Xylene	Chloroform	Ethyl Acetate
Adhesion	1						
Auto-aggregation	0.048	1					
Co-aggregation							
*S*. *aureus* ATCC 25923	0.048	0.734 **	1				
*E*. *coli* ATCC 25922	0.113	0.879 **	0.634 *	1			
Hydrophobicity							
Xylene	0.028	0.483	0.807 **	0.344	1		
Chloroform	−0.106	0.570 *	0.887 **	0.489	0.934 **	1	
Ethyl acetate	−0.163	−0.183	0.418	−0.234	0.716 **	0.672 **	1

*: *p* < 0.05, **: *p* < 0.01, bold values indicated significantly positive correlation.

**Table 3:** Antimicrobial activity of the probiotic strains against indicator pathogens.

Strains	*R* Value (mm)	
	*S*. *aureus* ATCC 25923	*E*. *coli* ATCC 25922
*L*. *plantarum* LC27	11.33 ± 0.76 (High)	14.17 ± 0.29 (High)
*L. reuteri* NK33	6.17 ± 0.76 (High)	8.83 ± 0.58 (High)
*L. rhamnosus* NK210	11.67 ± 0.29 (High)	14.00 ± 0.50 (High)
*B. longum* NK46	7.33 ± 0.29 (High)	9.83 ± 0.29 (High)
*B. bifidum* NK175	6.17 ± 0.29 (High)	8.33 ± 0.58 (High)

The scores of growth inhibition of the indicator strains are shown in parentheses. *R*: zone of clearance.

**Table 4:** Minimum inhibitory concentrations (MICs) of probiotic strains cell-free supernatants against tested indicator pathogens.

Strains	MIC (mg/mL)	
	*S*. *aureus* ATCC 25923	*E*. *coli* ATCC 25922
*L*. *plantarum* LC27	20	20
*L. reuteri* NK33	30	30
*L. rhamnosus* NK210	20	30
*B. longum* NK46	30	30
*B. bifidum* NK175	50	50

## Data Availability

The original contributions presented in the study are included in the article/Supplementary Material, further inquiries can be directed to the corresponding author.

## Supplementary Materials

The following supporting information can be downloaded at https://www.mdpi.com/article/10.3390/foods13142301/s1, Figure S1: Relationship between auto-aggregation (%) and cell surface hydrophobicity (%) of *Lactobacillus* strains.; Figure S2: Antimicrobial activity of the probiotic strains against (a) *S*. *aureus* ATCC 25923 and (b) *E*. *coli* ATCC 25922 by agar overlay method.
