# Supplementary material for: In Vitro Evaluation of Probiotic Properties and Anti-Pathogenic Effects of Lactobacillus and Bifidobacterium Strains as Potential Probiotics
Source: Foods. 2024 Jul 22;13(14):2301. doi: 10.3390/foods13142301 (PMC11276478; doi:10.3390/foods13142301)
Supplement: Supplementary file 1 [file foods-13-02301-s001.zip › foods-3111037-supplementary.pdf]

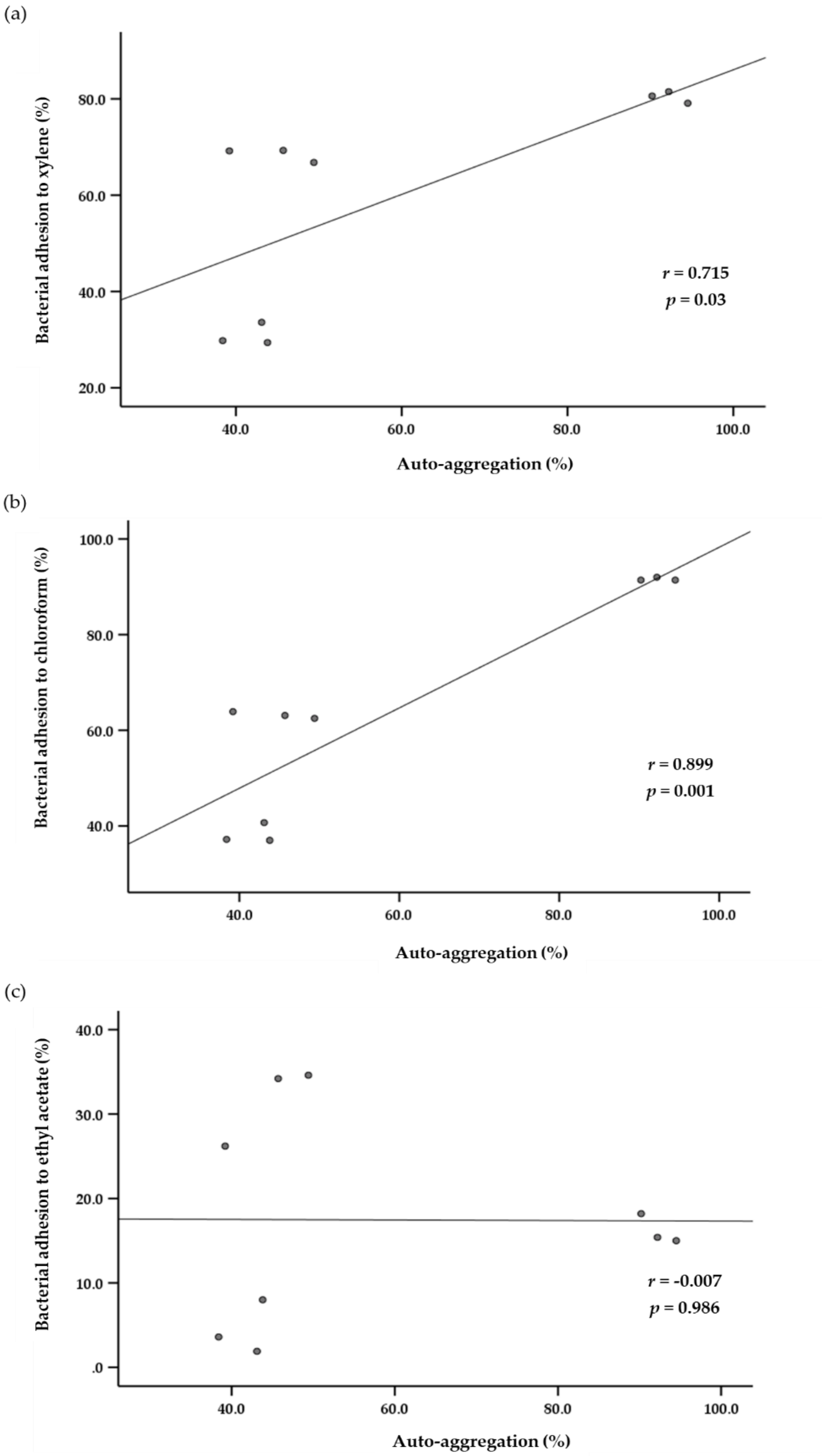

Figure S1. Relationship between auto-aggregation (%) and cell surface hydrophobicity (%) of *Lactobacillus* strains. (a) xylene, (b) chloroform and (c) ethyl acetate. r and p-value indicate Pearson's correlation and its significance, respectively.

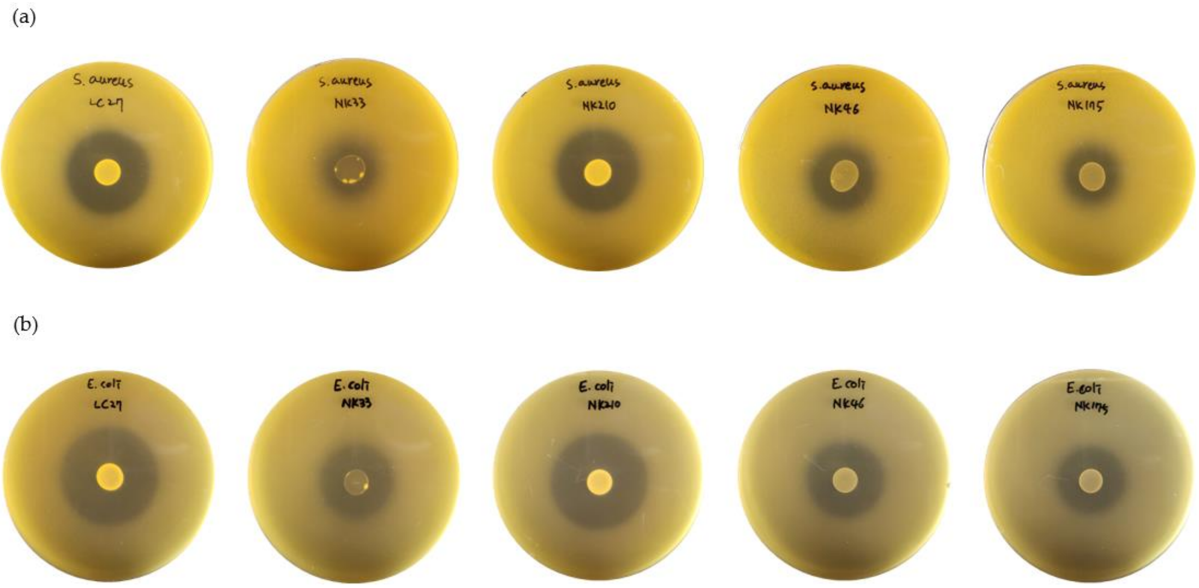

Figure S2. Antimicrobial activity of the probiotic strains against (a) *S. aureus* ATCC 25923 and (b) *E. coli* ATCC 25922 by agar overlay method.
